# The performance of the IES-R for Latinos and non-Latinos: Assessing measurement invariance

**DOI:** 10.1371/journal.pone.0195229

**Published:** 2018-04-03

**Authors:** Jitske Tiemensma, Sarah Depaoli, Sonja D. Winter, John M. Felt, Holly M. Rus, Amber C. Arroyo

**Affiliations:** Psychological Sciences, University of California, Merced, CA, United States of America; Fordham University, UNITED STATES

## Abstract

Violent acts on university campuses are becoming more frequent. Enrollment rates of Latinos at universities is increasing. Research has indicated that youths are more susceptible to trauma, particularly Latinos. Thus, it is imperative to evaluate the validity of commonly used posttraumatic stress measures among Latino college students. The Impact of Event Scale-Revised (IES-R) is one of the most commonly used metrics of posttraumatic stress disorder symptomatology. However, it is largely unknown if the IES-R is measuring the same construct across different sub-samples (e.g. Latino versus non-Latino). The current study aimed to assess measurement invariance for the IES-R between Latino and non-Latino participants. A total of 545 participants completed the IES-R. One- and three-factor scoring solutions were compared using confirmatory factor analyses. Measurement invariance was then evaluated by estimating several multiple-group confirmatory factor analytic models. Four models with an increasing degree of invariance across groups were compared. A significant χ^2^ difference test was used to indicate a significant change in model fit between nested models within the measurement invariance testing process. The three-factor scoring solution could not be used for the measurement invariance process because the subscale correlations were too high for estimation (*r*s 0.92–1.00). Therefore, the one-factor model was used for the invariance testing process. Invariance was met for each level of invariance: configural, metric, scalar, and strict. All measurement invariance testing results indicated that the one-factor solution for the IES-R was equivalent for the Latino and non-Latino participants.

## Introduction

As public violence continues becoming a mainstay in the United States [1], attention has turned to assessing its psychological impact. Of particular interest is the influence of campus violence. University campuses have been the venue for some of the deadliest attacks in recent years (e.g., Northern Illinois University, Virginia Tech, & University of California Santa Barbara), and thus the focus of extensive research [2–4]. Response efforts, including counseling and treatment, depend on accurately assessing and understanding the impact of such traumatic experiences. As such, research evaluating the validity of measures for psychiatric symptoms resulting from trauma is critical [5]. The present study aimed to assess measurement invariance of a commonly used posttraumatic stress symptomology scale between Latinos and non-Latinos.

### The Impact of Event Scale-Revised (IES-R)

The Impact of Event Scale-Revised (IES-R) is one of the most commonly used metrics for assessing posttraumatic stress symptomology [6, 7]. Although the scale has shown validity across different types of trauma (e.g., school shootings, [8, 9], the September 11^th^ terrorist attacks [10], abuse [11–13], and natural disasters [14, 15]), it is largely unknown if the IES-R measures the same construct across different cultural sub-samples (e.g., Latino versus non-Latino).

The Impact of Event Scale (IES; [16]) was proposed to contain two subscales, *intrusion* and *avoidance*. While some studies found support for this factor structure (e.g., [17, 18]), others identified a third factor: *sleep disturbance* [19]. In addition, one study identified a four-factor structure that included the factors *intrusion*, *effortful avoidance*, *emotional numbing*, and *sleep disturbance*. With the introduction of the DSM-IV [20], the IES was updated to include a *hyperarousal* subscale [6, 7]. The factor structure of the new scale, the IES-Revised (IES-R), has also received attention. In general, studies do not find strong support for the three proposed subscales (e.g., [15, 21–22]). This is in line with literature reviews reporting that PTSD is better characterized using a four-factor or even a five-factor structure instead of the three factors proposed by the DSM-IV [23, 24]. Indeed, King et al. [25], found that a four-factor solution represented data best. The four subscales that they confirmed were *intrusion*, *avoidance-numbing*, *hyperarousal*, and *sleep issues*. In contrast, Arnberg et al. [15] found that a five-factor structure provided the best fit in a sample of natural disaster survivors in Sweden. The five factors they identified were *intrusion*, *avoidance*, *numbing*, *dysphoric arousal*, and *anxious arousal*. However, even though these studies show that a four- or five-factor model should be preferred over either the total score or the three subscales, to the best of our knowledge, only one application of these models exist beyond King et al’s [25] and Arnberg et al.’s [15] papers. Specifically, Wang, et al. [14] replicated the four-factor model reported by King et al. [25] in a sample of Chinese earthquake victims.

Instead, the applied literature tends to focus on the total score of the IES-R [e.g., 9, 12, 13,26–30] or the three subscales (e.g., [9, 11, 31]). Thus, the current study will aim to examine these two factor solutions, so as to increase the potential generalizability of the findings to other studies.

In addition to research focusing on the factor-structure of the IES-R, some studies also examined the measurement invariance of the IES-R across groups or across time. For example, after King et al. [25] selected the four-factor model for the IES-R, they assessed measurement invariance for a U.S. undergraduate sample and an Israeli emergency room sample. They also assessed measurement invariance over multiple occasions for the latter sample. In both cases, support was only found for configural invariance, indicating that mean scores cannot be meaningfully compared across samples or across time. Wang et al. [14], using the same factor-structure, assessed measurement invariance for male and female participants. They found support for strict invariance across the two genders, indicating support for the comparison of mean scores across genders. Finally, Arnberg et al. [15] examined measurement invariance of their five-factor model across three time points. They found support for metric invariance across time, indicating that mean scores cannot be meaningfully compared across time.

### Measurement invariance across ethnic groups

The United States Surgeon General recently highlighted the importance of measuring the same construct across ethnic groups [32]. This recommendation is rooted in the notion that cultural factors affect how individuals define, evaluate, and approach their mental health problems. For example, an observed mean difference between groups may be caused by true differences across those groups on a mental health outcome. However, it is also possible that these differences are the result of differential interpretation of items across the groups; i.e., the items may preform differently across groups, thus tapping into a different underlying construct.

Ethnic minorities have been under-represented in clinical and health research [33]. This includes research involving the construction and validation of commonly used metrics in the field of psychology, which has been predominantly conducted on Caucasian samples [34]. The absence of ethnic makeup reporting of participants in research highlights the lacking concern over cross-cultural scale validation. The year the IES-R was published, only two-fifths of published research in the US reported the ethnicity of its participants [35], exemplifying the assumption that psychopathology is the same across cultures. Research has since demonstrated distinct cultural differences in psychopathology between Latinos and non-Latinos [36–41].

One major limitation of previous research on the factor-structure and potential measurement invariance of the IES-R, is that none of the previous studies have explicitly focused on Latino versus non-Latino sub-samples. In fact, studies neglect to report on sample ethnicity [21] or have included low numbers of Latino participants (e.g., [22, 25]). Failing to account for ethnic diversity when using such measures can lead to a host of errors including increased residual variance and increased Type II error rate [42, 43]. Research should use measures that have been created, validated, or adjusted for the population of interest [32, 43]. Still, the IES-R has not been validated with Latino samples.

As university campuses become more ethnically diverse, Latinos are showing the fastest rate of enrollment growth [44]. Latinos have also shown a greater risk of developing PTSD [45–48] and may experience more severe symptoms [36]. While the disparity in PTSD among Latinos could be due to the inappropriate use of culturally insensitive measures, it could also be due to differences in culture. Research indicates that the Latino population generally has been “neglected, misunderstood, or inappropriately served” by the mental health system [38]. Falicov states that mistreatment of Latinos in health care settings is rooted in cultural misunderstanding [40]. One study found that 28% of Latinos, in comparison to 5% of whites, felt that they were mistreated by a health care provider because of their ethnic background [49].

In addition, more inherent cultural factors could contribute to the disparity in PTSD. Research suggests that compared to non-Latinos, Latinos experience greater wishful thinking and self-blaming coping strategies, less social support, and greater perceived racism, which are all predictors of PTSD [36]. Latinos also experience greater peritraumatic dissociation following a trauma, which predicts subsequent PTSD onset [50].

Although measurement invariance on the IES-R has not been assessed with Latinos, research on other PTSD scales have found measurement invariance [48] and non-invariance [51] between Latino and Caucasian sub-samples. This inconsistency might be caused by study characteristics, such as the specific scale assessed in the study. Contractor et al. [48] examined the invariance of the UCLA PTSD-RI [52] while Hoyt and Yeater [51] examined the invariance of the PTSD Checklist—Civilian Version (PCL-C; [53]). In addition, this inconsistency may have been caused by important differences between the two study samples assessed. Contractor et al. [48] focused on children and adolescents who experienced a traumatic event, while Hoyt and Yeater [51] examined a sample of undergraduate students and did not explicitly assess whether or not the students had experienced a traumatic event. The inconsistency in substantive findings related to other posttraumatic stress scales warrants further investigation of the utility of the IES-R for Latinos and non-Latinos. It remains unclear if discrepancies in prevalence rates of PTSD among Latinos are due to inherent cultural differences or to scale-dependent, differential measurement of symptoms.

### Aim of the study

The Latino population in the United States is projected to increase 115% by 2060, making up one-quarter of the total United States population [20]. Given the increasing rate of trauma on college campuses [1, 54], the susceptibility of youths to trauma [5, 55–57], particularly Latinos [45–48] and the growing rate of Latinos at college campuses [44], it is imperative to evaluate measurement invariance of the IES-R.

The sample of the current study were all undergraduate students at a Hispanic serving institution located in Central California. The sample was taken following a traumatic event at their university. The current study aimed to assess measurement invariance for the IES-R between Latino and non-Latino college students.

## Materials and methods

### Measure

The Impact of Event Scale-Revised (IES-R; [6]) is a 22-item, self-report Likert-type measure that assesses posttraumatic stress symptoms on a scale from 0 (*not at all*) to 4 (*extremely*) in relation to a specific event. Respondents report how distressing certain difficulties related to the event have been over the past seven days (e.g., “I tried not to think about it.”). Participants in the current study responded to the scale with respect to the violent campus attack described below.

Scoring of the IES-R includes a total score (ranging from 0–88) and three subscales reflecting avoidance (e.g., deliberate efforts to avoid thinking or talking about the traumatic event), intrusion (e.g., thoughts or feelings about the traumatic event arising without conscious effort), and hyperarousal (e.g., an exaggerated startle response, angry outbursts, hypervigilance); these subscales correspond with the DSM-IV definition of post-traumatic stress [20]. Higher total (or subscale) scores reflect higher levels of distress.

While the IES-R is not generally used to diagnose PTSD, cut-off scores for a preliminary diagnosis have been proposed. Scores above 24 reflect significant clinical concern [58], scores above 33 reflect a probable diagnosis of PTSD [21], and scores above 37 reflect long-term suppression of immune system functioning [59]. High levels of internal consistency on the total score have been established across various samples (Cronbach’s α = .95-.96; 26, 27, 28). Likewise, internal consistency in the current sample was high (Cronbach’s α = .95).

As the applied literature focuses on the total score [e.g., 9, 12, 13,26–30] or the three subscales [e.g., 9, 11, 31], the current study will focus on examining these two factor solutions. Taking this approach increases the potential generalizability of the findings to other studies.

### Participants

Participants were 552 undergraduate students enrolled at a designated Hispanic-serving institution in Central California at the time of a campus stabbing. In November of 2015, an undergraduate university student stabbed four victims and fled through campus before being shot and killed by campus police. None of the victims were fatally injured; however, many students were exposed to traumatic scenes during and immediately following the attacks.

The current data come from a larger study looking at social media use in response to the attacks. Given growing interest in the psychological aftermath of campus violence [8, 9, 60, 61], and the increasing use of social media in response to public trauma [62–64], survey data on the role social media played in response to the attacks was collected five months later (note that due to an ongoing Federal investigation, data could not be collected until this time). The scope of the current study is to assess measurement invariance of the IES-R; as such, results on social media use and trauma are presented elsewhere (data not yet published). All study procedures were approved by the University’s Institutional Review Board, and all participants provided written informed consent prior to participating.

Participants were recruited with a listing for a study on social media use in response to the stabbings posted on the campus online research participation system. All participants self-identified as active Facebook users and received course credit for participating. Using the same method of administration, all participants completed an online survey which included the English version of the IES-R, as well as measures of depression, social support, and social media use in relation to the attacks. Of the 552 participants, seven (1.3%) were excluded from analyses for the following reasons: participant did not provide information on their ethnicity (*n* = 1), participant did not answer any of the IES-R questions (*n* = 3), and participant only answered a portion of the IES-R questions (*n* = 3). The final sample included 545 participants.

The majority of the sample (53%, *n* = 286) was present on campus at the time of the attacks, while 17% (*n* = 90) of these participants witnessed something related to the attacks. Approximately 20% (*n* = 110) of participants scored within the range of significant clinical concern on the IES-R (≥ 24), which is higher than the average rate of PTSD (6% - 9%) in college students [65, 66].

Average age of participants was 19.78 years (SD = 1.93 years). The majority of participants were female (73%, n = 399) and underclassmen (65%, n = 354). Fifty-six percent (n = 306) of the sample self-identified as Hispanic/Latino from a list of forced-choice options, while the remaining 44% self-identified as non-Latino (i.e., African American, Native American, Asian/Pacific Islander, Bi-racial, Caucasian, or other). See Table 1 for full racial and ethnic background of the sample.

**Table 1:** Racial and ethnic breakdown.

Race/Ethnicity	Frequency	Percent
Hispanic/Latino	309	56.2
Asian/Pacific Islander	120	21.7
Caucasian/White	46	8.3
African American/Black	32	5.8
Bi-Racial	26	4.7
Native American/American Indian	1	0.2
Other	16	2.9

Seventy-one percent of the sample identified as being first-generation college students. Although information on immigration and legal status was not collected in this study, approximately 8% of the University’s student population is undocumented [67, 68].

### Data analytic strategy

Data can be found in S1 Data. All models were estimated using M*plus* version 7.4 [69] with a weighted least squares-mean and variance adjusted estimator (WLSMV) and theta parameterization. Given that the IES-R items are rated on a 5-point Likert-type scale, items were treated as categorical variables. Collinearity was evaluated through item correlations on the full sample of valid (i.e., non-missing) responses. No problematic levels of collinearity were detected. Two questions reflecting sleep issues (items 2 and 15) were highly correlated (*r* = .82), but this level of correlation was not severe enough to cause problems in the invariance phase. Therefore, all models were estimated with the full set of items.

Multiple-group Confirmatory Factor Analysis (MGCFA) is used to formally test whether a scale is measurement invariant (MI), that is, whether latent mean scores can be meaningfully compared across groups. MGCFA uses a stepwise model comparison approach where each subsequent model restricts more parameters to be equal across groups [70]. The first step is to examine the factor-structure of the IES-R within each sample. For this purpose, one- and three-factor solutions were compared using confirmatory factor analyses (CFAs). The IES-R is typically scored as a three-factor scale, but researchers can also interpret the total score using a one-factor solution. These two options were explored to identify the most useful scoring method to implement within the measurement invariance testing process. For the one-factor model, all items loaded onto a general PTSD factor. The three-factor solution represented the three symptom clusters: Intrusion, Avoidance, and Hyperarousal. This model was included because the IES-R was developed to accurately represent these three clusters of symptoms [71]. The best-fitting solution for each sample was identified and compared. If the same general factor-solution emerged in both samples, the first level of invariance, (1) configural invariance, would be established.

Further measurement invariance was then evaluated by estimating several MGCFA models. Each level of MI allows for the comparison of additional parameters across groups. First, if metric invariance can be established, then covariances and regression coefficients can be compared across groups [72–73]. Scalar invariance permits the comparison of latent means across groups [73]. Finally, strict MI indicates that groups can be treated as one and the same. In other words, the construct is being measured in the exact same way across groups.

Following the guidelines prescribed by Meredith [70], four models were estimated and compared with an increasing degree of invariance across groups: (1) configural invariance within the MGCFA, where all parameters were allowed to vary across groups. For identification of the model, the factor means were fixed at zero and the factor variances and residual variances were fixed at one; (2) metric invariance, where factor loadings are constrained to be equal across groups, which also allows for the estimation of the factor variance in the second group; (3) scalar invariance, where factor loadings and thresholds are constrained to be equal across groups, and the factor mean and variance of the second group are estimated freely; and (4) strict invariance, where residual error variances are constrained to be equal across groups. Because these residual error variances were constrained in the configural, metric, and scalar models, a less stringent scalar model was estimated where the error variances were not constrained between groups. Goodness-of-fit indices were then compared to the original scalar model. As a final step, the latent factor means can be compared across groups.

Several model fit indices are reported in the analysis section. The robust χ^2^ difference test (*p* < .05; [33]) was used to compare the two different scoring options and different levels of invariance. As models were estimated with the WLSMV estimator, we used the DIFFTEST command in Mplus to estimate the robust χ^2^ difference test. The comparative fit index (CFI; for use in invariance testing, see [74]), Tucker Lewis fit index (TLI; [75]), and root mean square error of approximation (RMSEA; [36]) were used to examine the absolute fit of the scoring options. These measures were included because the χ^2^ test is known to be sensitive to minor divergences from invariance with larger samples [37–38]. With regard to comparing model fit of different levels of invariance, we also examine ΔCFI. Cheung and Rensvold [74] found that a ΔCFI smaller than or equal to -0.01 indicates that the null hypothesis of invariance should not be rejected (i.e., the more restrictive model should be retained).

## Results

Latino participants did not significantly differ from non-Latino participants on sum-scores of the IES-R total or on the three hypothesized subscales: Intrusion, Avoidance, and Hyperarousal (see Table 2). While there were no differences in the means of the two subsamples, Table 1 does show that a higher maximum score of the IES-R total and Intrusion subscale are observed for the Latino participants. In contrast, a higher maximum score of the Avoidance and Hyperarousal scale were observed for the non-Latino participants. To further understand the distribution of IES-R total scores in the two subsamples, we investigated the number of participants in each sample that scored at or above the clinical cut-off score of 24. Fifty Non-Latino participants (21%) score at or above the clinical cut-off while sixty of the Latino participants (20%) score at or above this cut-off of 24 point. This difference in prevalence of clinically high scores was not significant (χ^2^(1) = 0.07, *p* = .786, φ = .02).

**Table 2:** Descriptive statistics, t-statistics, and Cohen’s D comparing Latino and Non-Latino participants on IES-R scales.

	Latino (*N* = 306)				Non-Latino (*N* = 239)				*t*(*df* = 454)	Cohen’s *d*
	*M*	*SD*	*Min*	*Max*	*M*	*SD*	*Min*	*Max*		
IES-R Total	13.31	14.51	0	72	12.51	15.56	0	66	0.62 *ns*	0.05
IES-R Intrusion	3.97	5.48	0	30	3.89	5.65	0	24	0.18 *ns*	0.02
IES-R Avoidance	6.65	6.04	0	26	5.80	6.50	0	29	1.57 *ns*	0.13
IES-R Hyperarousal	2.71	4.09	0	20	2.82	4.39	0	21	0.31 *ns*	0.03

In addition to comparing the IES-R scores across the two subsamples, we also examined whether witnessing anything related to the attack was associated with higher total IES-R scores. The ninety participants who observed something related to the attacks were compared to the 455 participants who did not observe anything related to the attacks. The difference in IES-R score between the groups was significant, *t*(543) = -3.15, *p* = .002, Cohen’s *d* = 0.36. Cohen’s *d* indicates that the mean of the witness group (*M* = 17.46, *SD* = 18.74) was about one-third of a standard deviation above the mean of the non-witness group (*M* = 12.07, *SD* = 13.96). To ensure that the distribution of participants who had witnessed something related to the attacks was equal across the two subsamples, we examined whether Latinos or non-Latinos were more likely to witness anything related to the attacks. Thirty-five Non-Latino participants (15%) and fifty-five Latino participants (18%) were a witness. This difference in number participants who witnessed something was not significant (χ^2^(1) = 0.85, *p* = .356, φ = .04).

### Model comparison

Table 3 contains the results for the one- and three-factor solutions specified for the two subsamples. Both scoring options fit the data well, with a difference in CFIs less than 0.01 (see Table 3), which indicates comparable model fit across the scoring methods for Latino and non-Latino participants [34]. Although the CFIs were comparable, the χ^2^ difference test indicated that the three-factor solution fit the data significantly better than the one-factor solution (Δ χ^2^ (3) = 57.54, *p <* .05 for Latino, and Δ χ^2^ (3) = 63.79, *p <* .05 for non-Latino). However, further investigation into the three-factor solution for each subsample revealed that the subscales were highly correlated; factor correlations ranged from 0.92 to 1.00. This pattern was also present when the three-factor solution was estimated for the total sample, with factor correlations ranging from 0.92 to .99. Measurement invariance testing could not be implemented on this scoring solution due to a non-positive definite matrix resulting from the high correlations. High correlations such as these have been found in previous research on the IES-R [21, 31].

**Table 3:** Single sample CFA fit indices.

	Χ^2^ (df)	Δ Χ^2^ (df)	CFI	TLI	RMSEA
**Non-Latino**					
Three-factor	465.18 (206)*		.986	.984	.073 (.064 –.081)
One-factor	537.55 (209)*	57.54 (3)*	.982	.980	.081 (.073 –.090)
**Latino**					
Three-factor	590.43 (206)*		.971	.968	.078 (.071 –.086)
One-factor	651.14 (209)*	63.79 (3)*	.967	.964	.083 (.076 –.090)

* *p* < .05

*Note*: CFI = comparative fit index. TLI = Tucker Lewis fit index. RMSEA = root mean square error of approximation.

The one-factor solution produced standardized item factor loadings ranging from 0.51 to 0.92 (*R*^2^ values ranged from 0.26 to 0.85), indicating items loaded strongly on the single factor. In order to further assess whether the unidimensional structure is appropriate, we estimated a bifactor model as a follow-up analysis. The bifactor model, applied to the entire sample of Latino and non-Latino participants, fit the data well (χ^2^ = 684.53, RMSEA = .070, 90% CI = .064-.075, CFI = .982). The explained common variance (ECV) for the general factor was .92, which indicates that the IES-R is sufficiently unidimensional to warrant a one-factor model [76, 77]. Thus, the invariance testing process was conducted on the single factor (i.e., total score) solution.

### Invariance test

Several multiple-group CFAs were estimated to investigate the measurement invariance of the single-factor IES-R across Latino and non-Latino participants. Full results for the invariance test are presented in Table 4. This table includes two models (model 3 and 4b) that are equivalent to each other. We included both these models in the table and our analyses to ensure that we did not assume a stronger level of invariance than is supported by the data simply because we could not directly compare model 4a (conventional scalar invariance) to model 2 (metric invariance). The categorical nature of the indicators necessitates that the residual variances in the metric invariance model are fixed across groups in order for the model to be identified. This additional constraint prevents us from directly comparing the model fit between the metric invariance model and the conventional scalar invariance model (in which residual variances are freely estimated), because they are no longer nested. Thus, we were required to compare the metric invariance model to a much more restrictive strict invariance model (model 3). Finally, in order to ascertain that the strict model accurately reflects the patterns in the data, we also compared the strict invariance model to the conventional scalar invariance model, as these two models are nested.

**Table 4:** Fit indices for models testing various levels of measurement invariance.

	Χ^2^ (df)	Δ Χ^2^ (df)		CFI	ΔCFI	TLI	RMSEA
1. Configural	1175.56* (418)			.976		.974	.082 (.076 - .087)
2. Metric	869.99* (439)	1 vs. 2	29.036 (21)	.986	.010	.986	.060 (.054 - .066)
3. Scalar (actually Strict)	945.20* (526)	2 vs. 3	101.91 (87)	.987	.001	.988	.054 (.049 - .060)
4a. Scalar with free residuals	1094.77* (504)			.981		.983	.066 (.060 - .071)
4b. Strict	945.20* (526)	4a vs. 4b	26.04 (22)	.987	.006	.988	.054 (.049 - .060)

* *p* < .05.

*Note*. It should be noted that model fit improves (sometimes only slightly) through the progression of some of the measurement invariance testing phases. This increase in the fit indices is likely due to the fact that fewer parameters are being estimated (due to the natural restriction of parameters during the testing phases), thus driving model fit higher.

Both the robust χ^2^ difference test and ΔCFI indicate that invariance was met for each level of invariance: configural, metric, scalar, and strict. Thus, strict measurement invariance was established on the IES-R between Latino and non-Latino students.

As strict measurement invariance was established, the latent factor means of the subsamples can now be meaningfully compared. With the current model specifications, the non-Latino participant group was selected as the reference group, fixing their factor mean to zero. IES-R factor mean differences were estimated between the non-Latino and Latino participant groups, and the difference was not statistically significant (*t*[454] = 1.78, *p* = .076). All measurement invariance testing results indicated that the one-factor solution for the IES-R was equivalent for the Latino and non-Latino participants.

## Discussion

The current study aimed to assess measurement invariance for the IES-R between Latino and non-Latino participants. Given the increasing rate of trauma on college campuses [1, 54], the susceptibility of youths to trauma [5, 55–57], particularly Latinos [45–48], and the growing rate of Latinos at college campuses [44], we felt it was imperative to evaluate measurement invariance of the IES-R. Our analyses showed that strict measurement invariance was established between Latino and non-Latino participants for the one-factor solution of the IES-R. All items on the IES-R were found to be unique and reliable indicators of the scale, and the IES-R was found in this research—as well as others (e.g., [11, 21, 22])—to be a reliable survey for trauma research. The measurement invariance testing process uncovered that the IES-R performs equivalently across Latino and non-Latino participants.

### Broader Implications

Comparing different scoring options for any assessment brings to question the substantive implications of that scoring process. In the case of the IES-R, the current investigation found that the unidimensional model (i.e., the total score model) was optimal for scoring. Although this finding is in contrast with scoring recommendations, it points to an overall assessment of trauma as being a sufficient indicator as compared to multiple subscales. Further, a potentially interesting finding of the current study is that, after establishing strict measurement invariance, the IES-R scores of the Latino participants did not differ significantly from the IES-R scores of the non-Latino participants. This finding might indicate that a traumatic event such as a campus attack evokes the same reaction regardless of individual differences among the participants. Future research could further explore this hypothesis.

### Limitations and future directions

One limitation of this study is that we did not directly assess levels of acculturation (e.g., country of origin, country of birth, preference of language spoken) or cultural identity. However, given that participants in this study completed the English version of the IES-R (and all study procedures were also in English) and are enrolled in an English-speaking university in the United States, less-acculturated Latinos were likely not part of the sample [78]. In addition, culturally specific values were not directly measured here. As a result, we do not know the *level* of acculturation for the participants, nor do we know when their family first moved to the United States. Both of these factors would be interesting additions to future work. A follow-up study with even more diverse samples that included less-acculturated participants who are less fluent in English may show differences in their interpretation and scoring patterns for the IES-R, especially if they completed the scale in their native language compared to English. Given that the presumed level of acculturation in our sample is relatively high, we are unable to generalize these findings to Latinos living outside of the United States.

Another important distinction to highlight about the current investigation is that we did not inherently compare people from different countries; we were interested in self-identified identity of students residing in the United States. An interesting next step to this work would be a direct comparison of residents from different countries, as country-level grouping might have a different impact on the IES-R scale interpretation. For example, residents of countries that are more individualistic (e.g., the United States or Canada) might interpret the IES-R differently compared to residents of countries with a more collectivist culture (e.g., Latin-American countries); for more information on these important cultural differences see Hofstede, Hofstede, and Minkov [79].

Furthermore, future studies may also include an investigation of potentially related moderators (e.g., level of trauma exposure, socio-demographic characteristics, and other culture-specific values). These additional aspects would likely highlight the dynamic impact of culture in the study of trauma.

### Conclusion

Considering the growing Latino (college) population in the United States as well as elevated rates of PTSD in the Latino population, it is important to establish that the IES-R measures the same construct across participants from different ethnic backgrounds. This is especially important in light of the new political climate in the United States. The present study shows that, in the United States college population, the IES-R can be used to understand potential differences in posttraumatic stress symptomatology between Latino and non-Latino students. Therefore, future research will be able to rely on the IES-R for this purpose.

The IES-R appears to be a good resource for tapping into posttraumatic stress symptoms. Although the current investigation examined symptoms in students who recently experienced trauma on a college campus, we postulate that the findings from the invariance testing process are likely to generalize to other populations and trauma-based research. Ultimately, the IES-R appears to tap into the same constructs, in the same way, for Latino and non-Latino participants.

## Supporting information

S1 Data This is the S1 Data. (SAV)
